# LIVING LONGER WITH MODERATE AMOUNTS OF OXYGEN RADICALS IN FRUIT FLIES

**DOI:** 10.1093/geroni/igad104.2998

**Published:** 2023-12-21

**Authors:** Man Su Kim, Saurav Ghimire

**Affiliations:** Inje University, Gimhae, Republic of Korea; Inje University, Gimhae, Republic of Korea

## Abstract

The role of reactive oxygen species (ROS) in aging is complex and paradoxical. While the free radical theory of aging suggests that ROS cause cellular damage and senescence, some studies have shown that moderate ROS levels can enhance cellular adaptation and longevity. In this research, we used Drosophila as an in vivo model to investigate the optimal level of ROS that can promote healthy aging. We exposed flies to different concentrations of paraquat (PQ), a herbicide that generates superoxide anions, and measured their lifespan, stress resistance, and gene expression. We found that intermediate PQ levels (0.1 mM) extended lifespan and improved stress tolerance, while high PQ levels (0.5 mM) shortened lifespan and impaired stress response. We also observed an up-regulation of ROS scavenger genes, such as superoxide dismutase 1 and glutathione peroxidase, in the intermediate PQ group, suggesting a homeostatic mechanism to balance ROS levels. Our findings challenge the conventional view of ROS as harmful agents and provide novel insights into the molecular mechanisms of aging.

